# A Novel Role for *Mc1r* in the Parallel Evolution of Depigmentation in Independent Populations of the Cavefish *Astyanax mexicanus*

**DOI:** 10.1371/journal.pgen.1000326

**Published:** 2009-01-02

**Authors:** Joshua B. Gross, Richard Borowsky, Clifford J. Tabin

**Affiliations:** 1 Department of Genetics, Harvard Medical School, Boston, Massachusetts, United States of America; 2 Cave Biology Research Group, Department of Biology, New York University, New York, New York, United States of America; Stanford University School of Medicine, United States of America

## Abstract

The evolution of degenerate characteristics remains a poorly understood phenomenon. Only recently has the identification of mutations underlying regressive phenotypes become accessible through the use of genetic analyses. Focusing on the Mexican cave tetra *Astyanax mexicanus*, we describe, here, an analysis of the brown mutation, which was first described in the literature nearly 40 years ago. This phenotype causes reduced melanin content, decreased melanophore number, and brownish eyes in convergent cave forms of *A. mexicanus*. Crosses demonstrate non-complementation of the brown phenotype in F_2_ individuals derived from two independent cave populations: Pachón and the linked Yerbaniz and Japonés caves, indicating the same locus is responsible for reduced pigmentation in these fish. While the brown mutant phenotype arose prior to the fixation of albinism in Pachón cave individuals, it is unclear whether the brown mutation arose before or after the fixation of albinism in the linked Yerbaniz/Japonés caves. Using a QTL approach combined with sequence and functional analyses, we have discovered that two distinct genetic alterations in the coding sequence of the gene *Mc1r* cause reduced pigmentation associated with the brown mutant phenotype in these caves. Our analysis identifies a novel role for *Mc1r* in the evolution of degenerative phenotypes in blind Mexican cavefish. Further, the brown phenotype has arisen independently in geographically separate caves, mediated through different mutations of the same gene. This example of parallelism indicates that certain genes are frequent targets of mutation in the repeated evolution of regressive phenotypes in cave-adapted species.

## Author Summary

As we approach the 150th year since publication of *On the Origin of Species*, understanding the genetic architecture underlying evolutionary change remains an important challenge. When an organism enters a completely new environment or ecological niche, certain traits are no longer necessary for survival, while other new traits become critical for maintaining fitness. An example of such a transition is provided by cave animals. Many disparate taxa (e.g., crustaceans, salamanders, fish) have colonized caves, presumably to escape predation or expand populations into an unexploited niche. Strikingly, similar traits evolve convergently despite significant phylogenetic distance between these organisms. Caves provide a unique environment including the absence of light, few predators, few sources of food, etc. Under these conditions, one observes striking changes in morphology including reduction in eyes, expansion of non-visual sensory systems, and a suite of metabolic and behavioral changes. To understand the genetic underpinnings of these shifts, we have established the blind Mexican cave tetra, *A. mexicanus*, as a genetic system. In this paper, we use this system to investigate a classic morphological feature in these animals, depigmentation. We identify the gene *Mc1r* as being responsible for reduction in melanin content in multiple caves.

## Introduction

The blind Mexican cave tetra, *Astyanax mexicanus*, is a troglobitic characin fish exhibiting a variety of cave-specialized traits. In general, the cave ecosystem supports the evolution of some traits that are enhanced or increased over time (i.e., “constructive” traits), as well as some traits that decrease or degenerate over time (i.e., “regressive” traits) [1],[2]. It is important to note that the term “regressive” does not connote anything about whether the trait in question is more adaptive or whether its loss is selected, only that it is lost. Examples of constructive traits include enhanced chemosensory reception, e.g., increased number of taste buds and organs of the lateral line system [3]. Alternatively, examples of regressive traits include reduction of eye size and depigmentation [4]–[6].

**Figure 1:**
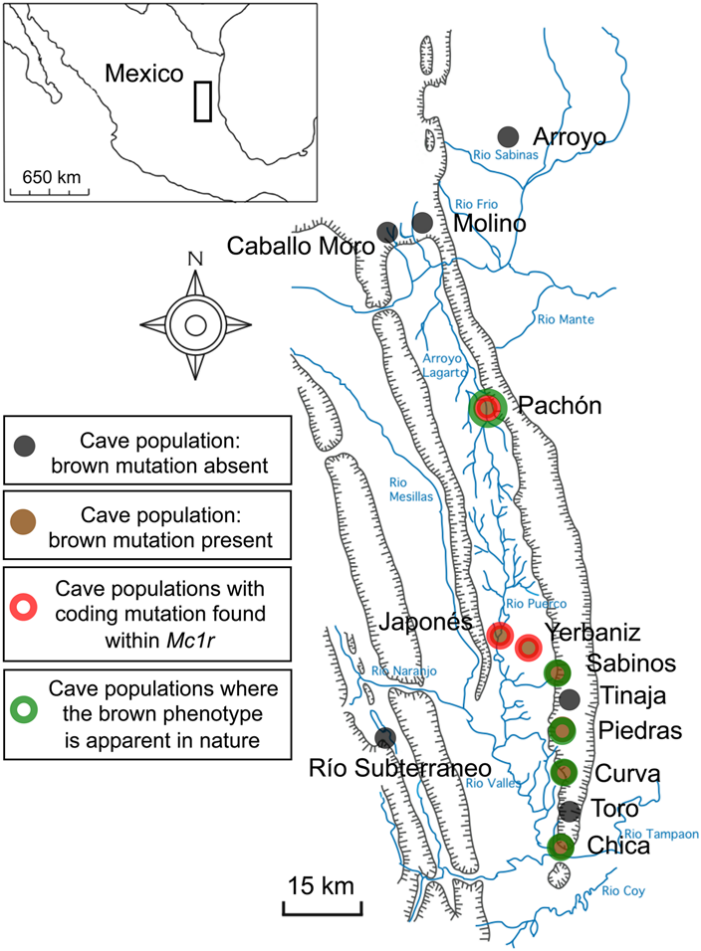
Schematic map and localities of cave and Surface populations of the Mexican tetra, *Astyanax mexicanus*. Several cave and Surface populations have been described throughout the Sierra de el Abra region of northeastern Mexico (inset). Cave populations in which the brown mutation is either absent (Molino cave) or has not been described (Tinaja cave) are depicted by a black dot. Cave populations in which the brown mutation has been described are denoted by a brown dot, while populations housing the brown mutation and carrying coding mutations in the gene *Mc1r* are depicted by a red/brown dot. Cave localities in which the brown phenotype is apparent in nature are labeled in green. Most localities do not harbor albinism (e.g., Chica, Curva, Piedras and Sabinos) and therefore, the phenotypic expression of the brown mutation in nature is inferred in these caves. Caves in which albinism has been reported include the Molino, Pachón, Yerbaniz and Japonés populations. Interestingly, of these caves only the Molino cave does not also harbor the brown mutation. While brown individuals have been reported in the Pachón cave [18], the same has not been reported in the literature in fish derived from the albino Yerbaniz/Japonés cave populations. Surface populations of *Astyanax* are found widely present throughout the rivers and streams of the region. Reports of the brown mutation and map of localities adapted from [4], [16], [18], [88]–[90].

At least 29 different cave populations from northeastern Mexico have been described, with Pachón cavefish being geographically isolated and cave-specialized (Figure 1) [7]–[9]. Members of each cave can be crossed with the Surface, sighted ancestral form to create viable F_1_ hybrids [10]. Crosses and trait distribution analyses in F_2_ individuals have demonstrated that several regressive traits, e.g. eye loss and depigmentation are polygenic [4], [11]–[15].

Among the most notable traits characterizing these fish is the marked reduction in skin pigmentation [4],[16], occurring independently in multiple cave forms [17]. While broadly defined, pigmentation in *Astyanax* is polygenic; some particular aspects of pigmentation are inherited in a monogenic, recessive fashion [4],[18]. As an example, albinism was recently discovered to be a monogenic trait caused by loss-of-function alleles of *Oca2*, this gene having been independently mutated in three different cave forms [1].

An additional simple trait affecting body pigmentation, termed the brown mutation, was described in the late 1960's as being present in several caves (Figure 1). The brown phenotype, affecting eye color as well as the number and size of melanophores on the body [18], was observed in the wild in fish from the Chica, Pachón and Sabinos caves. In addition, complementation test crosses carried out between F_1_ individuals derived from surface and various cave populations showed that the same locus was responsible for the brown phenotype in the Curva, Pachón, Piedras and Yerbaniz caves [16]. Three cave populations have been reported to harbor albinism mutations, including individuals in the Molino, Pachón and the inter-connected Yerbaniz and Japonés caves [1],[4],[16],[19]. As noted above, the brown phenotype also is found in two of these cave systems.

Further, in contrast to the Pachón cave, where the brown phenotype has been observed in individuals that do not carry the albino mutation, there is no published evidence that fish from the linked Yerbaniz/Japonés populations ever display the brown phenotype in nature. Therefore, it is not clear whether the brown mutation arose prior to the evolution of albinism in this population or, alternatively, if the brown mutation became fixed following the presence of epistatic albino mutations. Therefore, the cave populations that exhibit the brown mutation in nature, based on published data and/or inference through lack of albinism in these caves, include the Chica, Curva, Pachón, Piedras and Sabinos populations (Figure 1; green).

Laboratory crosses have been used to examine the inheritance of the brown phenotype. Segregation was analyzed in fish descended from a Surface×Pachón cave cross by scoring eye color of seven-day-old F_2_ larvae (derived from a cross of F_1_ hybrids of Surface and Pachón cavefish) as black, brown or pink (i.e., albino). When controlling for albinism, the frequency of individuals demonstrating the brown phenotype strongly predicted the participation of a single, recessive allele (black-eyed frequency = 0.73, brown-eyed frequency = 0.27, N = 5094) [18].

**Figure 2:**
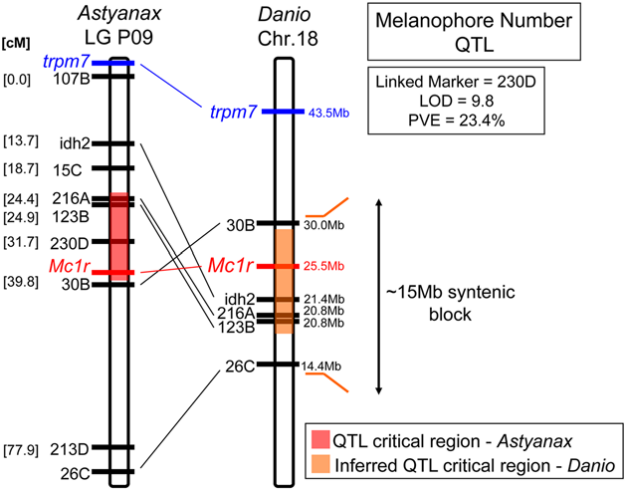
*Astyanax* linkage group P09 anchors strongly to *Danio* chromosome 18. Genomic sequences flanking microsatellites on *Astyanax* linkage group P09 are localized to within a ∼15 Mb stretch of *Danio* chromosome (Chr.) 18. Two candidate genes, *Mc1r* and *trpm7*, are present on chromosome 18 in *Danio*, however only *Mc1r* co-localizes to the critical region (red rectangle) of the melanophore number QTL on *Astyanax* linkage group P09. Legend: QTL critical region on *Astyanax* linkage group P09 (red rectangle), inferred QTL critical region on *Danio* chromosome 18 (orange triangle).

**Figure 3:**
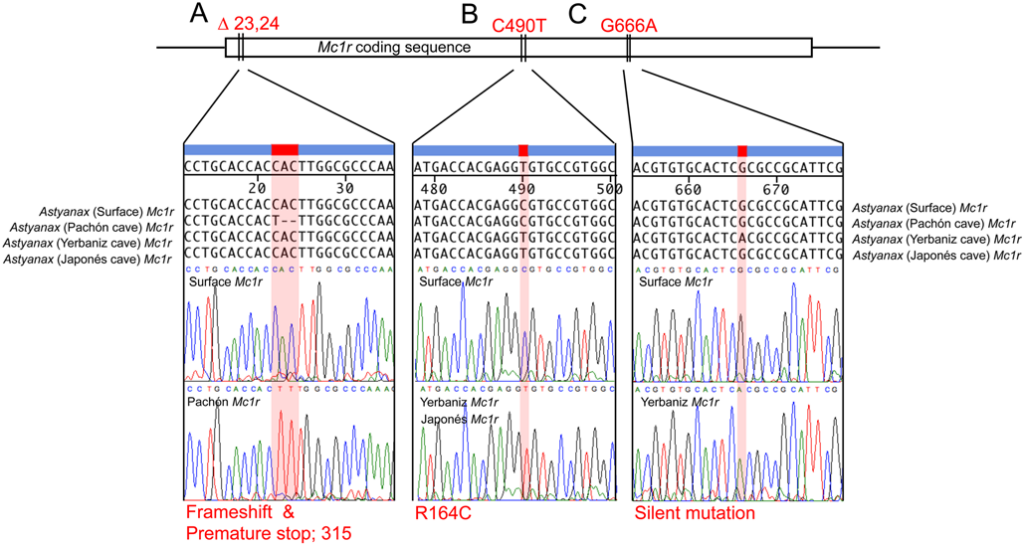
Sequence analyses of *Mc1r* open reading frame in Surface, Pachón, Yerbaniz and Japonés cavefish of *Astyanax mexicanus* reveal three coding mutations. A schematic representation of the *Mc1r* coding sequence is shown with each of three mutations identified in our sequence analyses. (A–C) Each of the three mutations is magnified to show nucleotide alignments for the Surface, Pachón, Yerbaniz and Japonés alleles of *Mc1r*. Below each alignment is the representative chromatogram sequence data for the Surface (wild type) compared with variant sequences. Three coding alterations were observed in three of a total nine caves completely sequenced for *Mc1r* in this study. (A) A 2-bp deletion at positions 23,24 was discovered in members of the Pachón cave population of *Astyanax*. This 2-bp deletion (red) causes a frame-shift and the introduction of a premature stop codon at nucleotide position 315. (B) We discovered an additional mutation at position 490 that causes an arginine to cysteine modification at amino acid position 164 (red). The identical mutation was discovered through analyses of both the Yerbaniz and Japonés populations, however these caves are likely members of the same cave population, possibly joined via a subterranean interconnection. (C) An additional silent mutation (G666A; red) was observed only in Yerbaniz individuals.

In this report, we investigate the genetic basis for the brown mutation by screening F_2_ individuals derived from an equivalent cross (Surface×Pachón cave hybrids) to that used in the original descriptions of this mutant [18]. We screened a pedigree of 488 individuals with 262 microsatellite markers, expanding upon pedigrees previously described [1],[2]. Consistent with other studies, our linkage analysis revealed a single, strong QTL influencing melanophore number in the post-optic region of the head and the dorsal flank in individuals derived from the Surface×Pachón cross (Figure 2). When we used the same criteria for mapping melanophore number in a Molino cave×Surface cross no statistically significant QTL were obtained. This is consistent with the reported absence of the brown mutation in this particular cave population (Figure 1) [16]. Using a candidate gene approach, we cloned and characterized the *Astyanax* form of the gene, *melanocortin type 1 receptor* (*Mc1r*), as the likely locus controlling this trait. Sequence analyses of the open reading frame (ORF) of *Mc1r* in Pachón individuals revealed a 2-base-pair deletion in the extreme 5′ end of the coding sequence, corresponding to the N-terminal domain of *Mc1r* (Figure 3A).

The Mc1r protein is a member of the GPCR superfamily of genes, comprised of an N-terminal domain, seven hydrophobic transmembrane domains, and a carboxy terminal domain [20]. One of the primary functions of Mc1r is to activate adenylyl cyclase in response to ligand binding, resulting in an intracellular increase in cAMP levels [21]. Mc1r binding leads to activation of downstream effectors in the pigmentation pathway, including the target gene *mitf*, which is transcriptionally upregulated by cAMP signaling in melanocytes [22]. Coding mutations in this gene have been described in model systems, including the classical ‘extension’ locus mouse mutant, which lacks normal functioning of Mc1r [23]. Coding sequence alterations are also known from natural populations, associating strongly with distinct coat and plumage color morphs in a variety of mammals and birds, respectively [24].

Depigmentation has arisen multiple times in different caves; therefore we extended our search for variant alleles to twelve other caves. We found an additional, independent mutation in the Yerbaniz cave (known to harbor the brown mutation) as well as Japonés cave individuals (Figure 3B,C). The point mutation present in these caves, C490T, alters an arginine residue homologous to that identified in certain human individuals with the red hair color (RHC) phenotype [25]–[28].

This analysis identifies a novel role for *Mc1r* in the evolution of degenerative phenotypes in blind Mexican cavefish. Further, we demonstrate that the brown phenotype has arisen independently in multiple forms of cavefish, mediated through different mutations of the same gene. This example of parallelism is consistent with other recent studies suggesting that certain genes may be frequent targets of mutation in the repeated evolution of similar phenotypes.

## Methods

### Crosses, Genotyping, and QTL Mapping

We used a previously described Pachón F_2_ pedigree obtained from a cross between the Surface×Pachón cave morphs of *Astyanax mexicanus* [1],[2]. Briefly, a Pachón individual was crossed to a Surface individual, and two sibling F_1_ individuals were crossed to produce 539 F_2_ individuals. 488 of these individuals were genotyped for *Mc1r*. All F_2_ progeny were raised with two individuals per tank (to control for size variation) and euthanized at 7 months of age. We performed an additional analysis of the melanophore number trait in a Molino backcross comprised of 111 individuals. This cross was performed by crossing a Molino cavefish to a Surface fish, and then mating an F_1_ individual to a second Molino fish. This backcross progeny set was reared in group tanks, euthanized at 14 months and fixed in 4% paraformaldehyde. Fin clips were collected from all individuals to isolate genomic DNA for subsequent genotyping. All *Astyanax* animal care protocols were approved by the NYU/University Animal Welfare Committee. Each individual was genotyped with 262 microsatellites using PCR reactions carried out in a 10 µl volume containing: 0.1 mM MgCl2, 6 mM Tris-HCl, pH 8.3, 30 mM KCl, 0.006% glycerol, 0.25 mM dNTP mix (Roche), 0.06% Tween, 0.06% Nonidet P-40, 0.25 units of Taq DNA polymerase (Roche), 5 nM forward primer, 200 nM reverse primer and 200 nM of the fluorescent tag primer: 5′-CACGACGTTGTAAAACGAC-3′ labeled with one of two phosphoramidite conjugates (Hex and Fam) and amplified using the PCR program previously described [1].

QTL mapping was carried out using the interval mapping function of MapQTL (version 4.0) to determine the LOD scores and percent variance explained (PVE) at the melanophore number locus using a permutation test, as previously reported [1]. *Mc1r* was placed on the linkage map using primers designed around an informative size-length polymorphism in the coding sequence (forward primer: 5′-TTCCTAAAGAGACCCCAGACC-3′; reverse primer: 5′-GCATTCATATCCCCCAGAGA-3′). Similarly, the gene *trpm7* was placed on the linkage map using the following primers designed around an informative size-length polymorphism in an intron (forward primer: 5′-TGCAGGCACTAAATATGCTACAA-3′; reverse primer: 5′-GATGGATAAAAAGGAGGTGAGG-3′).

Several additional cave and surface individuals collected from the wild were genotyped at the *Mc1r* locus (Table 1). Individuals were collected in the wild from the following cave localities: Arroyo, Caballo Moro, Chica, Curva, Japonés, Molino, Pachón, Río Subterraneo, Tinaja, Toro, Yerbaniz; and the following surface localities: Carolina, Honduras, Jutiapa, Mosquito Coast, Pantepec. Note that the Mosquito Coast and Jutiapa populations were drawn from the Atlantic drainage in Honduras; Carolina is in east central Oaxaca in Mexico in the Río Coatzacoalcos Atlantic drainage; and Río Pantepec is a tributary of the Río Tuxpan in Veracruz. In addition, individuals from an inbred strain of Piedras cavefish were analyzed. The extent to which cavefish and Surface fish at various localities are threatened is unknown. Therefore, genotyping was carried out in the most individuals we could sample from each locality given collection limits. Each individual was genotyped for the 2-bp deletion using size-length polymorphic primers (as above) and the C490T SNP (forward primer: 5′- ATGATCTGCAGTTCCGTGGT -3′; reverse primer: 5′-TCCGTGTGGTAGACGATGAA -3′; SNP primer: 5′-ACAGCATCATGACCACGAGG-3′) using the SNaPshot kit (Applied Biosystems).

**Table 1:** Summary of variant *Mc1r* genotypes collected from multiple cave and surface fish.

Locale	*Mc1r* genotype †	Number of fish genotyped
**Cave populations**		
Arroyo	- -	12
Caballo Moro	- -	9
Chica	- -	39
Curva	- -	13
Japonés	C490T; C490T∶490C; C490T∶Δ23,24	8; 2; 1
Molino	- -	10
Pachón	Δ23,24	15
Piedras	- -	5
Río Subterraneo		
Tinaja	- -	4
Toro	- -	3
Yerbaniz	C490T; C490T∶Δ23,24	9; 1
	**Number of cave individuals genotyped:**	140
**Surface populations**		
Arroyo Sarco	- -	16
Chamal	- -	7
Honduras	- -	129
Los Castros	- -	10
Micos	- -	9
Ocampo	- -	7
Río Choy	- -	7
Río Coatzacoalcos	- -	6
Río Frio	- -	7
Río Pantepec	- -	5
Río Tampaon	- -	10
Río Tantoan	- -	8
Sta Clara	- -	10
	**Number of surface individuals genotyped:**	231

**†:** Consensus (wild type) *Mc1r* genotype is denoted by - -.

### Phenotypic and Brown Mutant Analyses

Melanophore number was assayed in two distinct regions of non-albino Pachón F_2_ cross individuals as previously described [2],[15]. Both phenotypic measurements yielded the identical QTL, indicating that both measurements reflect the same genetic basis. We performed an analysis of melanophore number in three distinct regions of adult Molino backcross individuals. No QTL was detected in this pedigree, consistent with the lack of the brown mutant phenotype in fish derived from this cave [16].

Pigmentation differences were compared between individuals demonstrating the brown phenotype (i.e., those carrying two copies of the Pachón Δ23,24 allele) and surface (wild type) individuals. First, individual scales were carefully removed from the dorsal region of the fish, in the same region assayed in the original phenotypic analysis [2],[15]. Each scale was assessed for the number of melanophores and the amount of melanin per scale. In sum, 60 scales were collected from surface (n = 4) and brown mutant (n = 4) individuals of approximately the same age, washed briefly in physiological saline solution (130 mM NaCl, 2.7 mM KCl, 5.6 mM D-glucose, 1 mM EDTA, 5 mM Tris-HCl, pH 7.2) and fixed for several minutes at room temperature in 4% Paraformaldehyde (pH 7.4) as previously described [29]. Scales were whole-mounted on slides and imaged at various magnifications using either a Leica MZ FLIII stereoscope or Zeiss Axiophot compound microscope. All images were collected with the ACT-1 software program using identical lighting and software settings.

Mean melanin content for wild type versus brown phenotype melanophores was compared between 40× images of surface (wild type) and brown mutant individual melanophores. Photographs were light/dark-inverted using Adobe Photoshop CS3 and traced using the “freehand selection” tool in ImageJ 1.40 g (NIH). The mean pixel intensity value (ranging from 0 to 256) was collected for each inverted image (n = 16, each phenotype). All measures were tested for significance at the p = 0.01 level using a Student's t-test. All statistical analyses were carried out using Microsoft Excel (version X).

Two additional qualitative comparisons of pigmentation were performed. Cryosections of the dorsal flank from the identical regions of the surface and brown mutant individual were collected to compare sectioned melanophore morphology and melanin content. 20 µm thick sections were collected on Superfrost slides (VWR), incubated at room temperature for 1 hour, rinsed in PBS and mounted using gelvatol mounting medium. All images were collected (as above) using identical lighting and imaging software settings.

Ultrastructural analysis was performed using representative surface (wild type) and brown mutant tail tissue. Tissues were fixed and embedded in resin prior to ultrathin (95 nm) sectioning for electron microscopy analysis. Samples were analyzed with a Tecnai G^2^ Spirit BioTWIN scope at the Electron Microscopy Core Facility at Harvard Medical School using an AMT 2 k CCD camera.

### BLAST Analyses

We modified the method of Stemshorn et al., 2005 [30] to determine in the physical genome of *Danio rerio* the presumptive location of sequences homologous to the microsatellites we have identified in *Astyanax mexicanus*. Accordingly, we performed BLAST searches of the complete genomic DNA clone sequence from each of our microsatellites. All searches were carried out in Ensembl, assembly Zv7, release 47, using the following search parameters: E value cutoff: 10; Search sensitivity: No optimization; Search engine: BlastN; number of returned hits: 10. For each returned hit, we recorded the number of alignments, number of hits, chromosomal position, Stats score, E value, length of the identified sequence, percent of the sequence identified, additional hits, and whether the top hit was part of a coding or non-coding region. We assembled an ‘anchored’ version of the linkage group (Figure 2) containing the melanophore number QTL only using the strongest hits, as determined by the Stats score and E values.

### PCR Cloning of *Mc1r*

Total RNA was isolated and pooled from either 4 adult Surface individuals or 4 adult Pachón cavefish individuals using Trizol reagent (Invitrogen; Carlsbad, CA), according to manufacturer protocols. cDNA was generated from these pools using Transcriptor RT according to manufacturer protocols (Roche), and subjected to gradient PCR amplification. Using a degenerate cloning strategy, we designed primers against the following conserved amino acid residues: GLISLVENI (forward: 5′-GGGCCTGATCTCCCTGGTNGARAAYAT-3′) and IICNSLIDPL (reverse: 5′-GGGGGTCGATCAGGGAGTTRCADATDAT-3′) using the online primer design software CODEHOP [31] (blocks.fhcrc.org/codehop.html). We amplified a 736-bp fragment in both Surface and Pachón cave form cDNA template using a gradient PCR program (95°C for 2:00; 95°C for 0:30; 48°C–58°C for 0:30; 72°C for 1:30; cycle to Step 2, 34 times; 72°C for 10:00; 4°C). PCR fragments were subcloned into pGEM-Teasy vector (Promega; Madison, WI), sequenced, and identified using the NCBI BlastX search algorithm.

We extended our transcript sequence in the 3′direction using primers designed from known sequence in combination with the Smart RACE kit (Clontech) prepared using fresh total RNA from several representative Surface and Pachón cave individuals. Attempts to clone the 5′UTR using the same kit were unsuccessful, therefore we used a GenomeWalker kit (Clontech) to clone the 5′ end of *Mc1r*, using gene-specific primers designed using the online software program Primer3 (frodo.wi.mit.edu). The open reading frame of *Mc1r* was determined using the “search ORF” function in EditSeq 6.1 (DNASTAR Lasergene, version 6). The predicted amino acid structure was then compared to the known amino acid structure of closely related teleost fish and other vertebrates to infer correct Mc1r protein size.

Using primers designed to amplify full-length *Mc1r*, we cloned a 972-bp fragment of the ORF from Surface and genomic DNA derived from individuals from the following caves: Arroyo, Chica, Curva, Japonés, Micos, Molino, Piedras, Tinaja and Yerbaniz. We cloned a 970-bp fragment in genomic DNA derived from Pachón cave form individuals. Sequences were aligned and analyzed using the Clustal V method in MegAlign 6.1.2 (DNASTAR Lasergene, version 6).

### Morpholino Injection Experiments and Scoring

The morpholino, 5′-AGTGATGGCGCGAAGAGTCGTTCAT-3′, was designed to the first 25-bp of *Danio Mc1r* ORF sequence based on the accessioned sequence NM_180970 (NCBI). Morpholino injections were carried out using a variety of concentrations ranging from 0.2 µM to 1 µM in a 1 nl volume. All concentrations of morpholino injection resulted in decreased melanin, with correspondingly stronger phenotypes with increased concentration of morpholino injection. Several different concentrations were tested alone and in combination to determine the optimal concentrations and volumes for survival.

We injected single-celled embryos with 1 nl of 0.2 µM concentration of morpholinos either alone, or in combination with *in vitro* transcribed RNA derived from Surface, Pachón or Yerbaniz alleles of *Mc1r* using the mMessage mMachine kit (Ambion). All RNA injections were carried out in 1 nl injection volumes of 5 picomolar concentrations. Embryos were injected at day 0 and reared at ∼27°C. Phenotypes were assayed and scored every 24 hours. By the fifth day of development, we noted a decrease in the phenotypic penetrance, likely as a consequence of compensatory rescue from endogenous Mc1r activity. Therefore, we scored phenotypes of double-injected individuals at the fourth day post-fertilization, around hatching (prim-25, *Danio rerio*) [32]. Embryos were scored as having “normal” pigmentation corresponding to wild-type, uninjected individuals; or, “reduced” pigmentation that corresponded to the same phenotype as morpholino-alone injected individuals.

## Results

### Phenotypic Analysis of the Brown Mutation

Studies initially describing the brown mutation focused on its presence in the Pachón, Chica and Sabinos caves (Figure 1), near Tamaulipas, Mexico [8],[18]. More recently, the brown mutation was characterized in additional cave forms of *Astyanax* through a series of complementation test crosses carried out among several independently derived cave forms, including: Curva, Piedras, Molino and Yerbaniz caves (Figure 1) [16]. These studies demonstrate the presence of the brown phenotype in every cave studied with the exception of the Molino cave, in which skin pigmentation in F_2_ offspring did not differ from Surface form individuals (Figure 1) [16].

**Figure 4:**
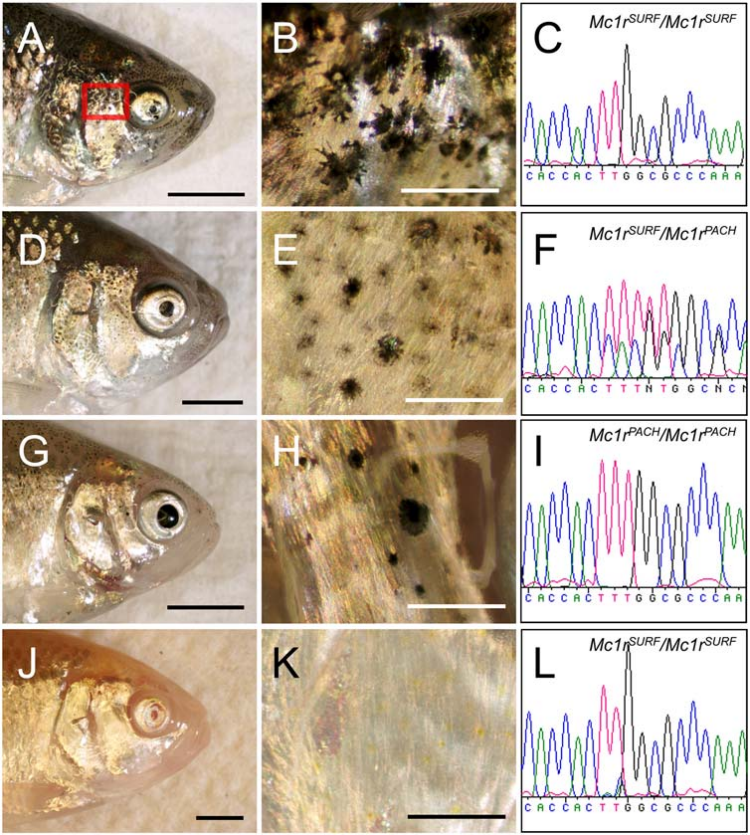
*Mc1r* genotype-phenotype correlation in representative members of an F_2_ pedigree derived from a Surface×Pachón cavefish cross. (A,D,G,J) We crossed two F_1_ hybrid individuals to generate a small pedigree of individuals demonstrating a range of pigmentation phenotypes. (A) Each fish was photographed from the right side to visualize the entire head or a region just posterior to the orbit of the eye (red rectangle). Each individual was also genotyped for the polymorphic region of *Mc1r* ORF housing the fixed 2-bp deletion found in members of the Pachón cave. (C,F,I,L) The resulting chromatogram sequence data is depicted to the right of each individual image. (A–C) We found that the darkest individual, with the most melanophores, carries two copies of the Surface form of *Mc1r*. (D–F) The heterozygous individual demonstrates roughly the same number of melanophores, however each cell appears to produce less eumelanin. (G–I) A non-albino F_2_ individual with two copies of the Pachón allele of *Mc1r* demonstrates the least amount of pigmentation compare to other pigmented genotypes, however this individual clearly differs from (J) albino phenotypes. (J–L) An albino individual carrying two mutant copies of the gene *Oca2* [1] who carries two Surface copies of *Mc1r* cannot produce pigment, demonstrating the epistatic nature of the albino mutation. Scale bars: A,D,G,J = 3 mm; B,E,H,K = 500 µm.

We presume each of the classical reports refer to the same trait, given that all describe identical manifestations of the brown mutant: diminished melanin content and decreased numbers of melanophores on the head and flank (Figure 4G) [4],[16],[18]. It should be noted, however, that Wilkens, 1988 [p. 307 [4]] describes the number of melanophores as an “additively polygenic” trait, indicating that additional genes may be relevant for the number of melanophores developing on the entire body, or different regions thereof. Nevertheless, consistent with our work, these authors report the existence of a recessive allele (the brown gene mutation) that “reduces the melanin content of the melanophores” in multiple cave forms through convergent evolution [p. 550 [16]].

**Figure 5:**
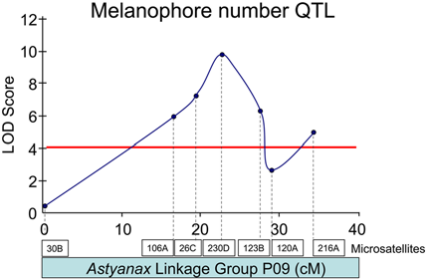
A melanophore number QTL resides on *Astyanax* linkage group P09. The genetic architecture of the melanophore number trait in our cross yields a single strong QTL consistent with the predictions of breeding experiments, as previously described [18]. The x-axis depicts the relative positions of microsatellite markers on linkage group P09, LOD score values (y-axis) across markers are depicted in dark blue. A LOD score significance value of 4.0 is depicted by a horizontal red line.

Our linkage studies were designed to allow us to address as many traits as possible in a single cross, and the stage at which the fish were assessed (see Methods) was chosen accordingly. Thus, we could not replicate the initial assay that identified the brown gene [18], i.e., fixing 7-day-old larvae and screening eye color. As an alternative, we counted the number of melanophores in a circumscribed region immediately posterior to the optic region (see Figure 4A; red box), and on the dorsal flank of fish (not shown) from the Pachón F_2_ cross. Both phenotypic analyses yielded the identical result, a single QTL on *Astyanax* linkage group P09 (Figures 2, 5), based on the linkage map published by Protas et al., 2008 [2]. This QTL had a LOD score of 6.6 for the dorsal melanophore count and 9.8 for the eye region melanophore count, each explaining 16.5% and 23.4%, respectively, of the phenotypic variance of this trait.

### *Astyanax* Linkage Group P09 Anchors Strongly to *Danio rerio* Chromosome 18

To accelerate our search for candidate genes that could mediate the effect of the brown phenotype QTL, we decided to “anchor” the linkage group encompassing our QTL for melanophore number to the *Danio rerio* physical genome. We adapted the method of Stemshorn et al., 2005 [30] to analyze the genomic DNA sequences flanking our polymorphic microsatellites via BLAST searches against the latest version of the *Danio rerio* physical genome (Ensembl Assembly Zv7, Sanger Institute; www.ensembl.org). For approximately 50% of the *Astyanax* linkage group P09 (LG P09) genomic markers analyzed, the Ensembl BLAST search algorithm identified unique *Danio rerio* sequences as plausible homologs (Figure 2). As described in Methods, these were obtained by only accepting hits with the lowest E values and highest Stats scores (indicating the highest support for sequence similarity between *Astyanax* and *Danio*). Using these criteria, the sequences homologous to *Astyanax* LG P09 are localized within a roughly 15 Mb stretch of chromosome 18, including markers flanking the brown QTL (Figure 2).

We also performed BLAST searches in all of the other available sequenced teleost genomes (data not shown), but found the highest number of significant hits were from *Danio rerio*. This likely reflects a more recent phylogenetic relationship between *Danio* and *Astyanax*, compared with other teleost model systems with a sequenced physical genome [33]. We could not exclude the possibility that the brown QTL itself might reside in a small genomic interval translocated from chromosome 18 to an entirely different genomic region in *Astyanax*. In spite of this formal possibility, the large ∼70 cM block of synteny between the genomes was suggestive enough that we developed a list of candidate genes within or near the syntenic critical region (Figure 2; orange) that could potentially affect the trait of interest (i.e., could decrease melanin and/or reduce numbers of melanophores) based on published activities.

Two genes residing on chromosome 18 in *Danio rerio* stood out, *transient receptor potential melastatin 7* (*trpm7*) and *melanocortin 1 receptor* (*Mc1r*). Size-length polymorphisms between the two morphotypes (intron of *trpm7*, coding region of *Mc1r*) were used as markers to place these genes on the *Astyanax* linkage map. *Trpm7* was indeed placed on *Astyanax* LG P09, however its position is over 25 cM from the melanophore number QTL (Figure 2). In contrast, *Mc1r* was positioned directly at the QTL for melanophore number with a peak LOD score, making *Mc1r* our strongest candidate gene for the melanophore number QTL in *Astyanax*. We also cloned several other *Astyanax* genes involved in pigmentation in model organisms that did not lie on *Danio* chromosome 18, including *melanin concentrating hormone receptor 1a* (*mch1ra*), *kit receptor* (*kita*) and *shroom2*. As predicted, these genes mapped outside LG P09 in *Astyanax*.

### Sequence Analysis of *Mc1r* in Surface and Cave Forms of *Astyanax*

**Figure 6:**
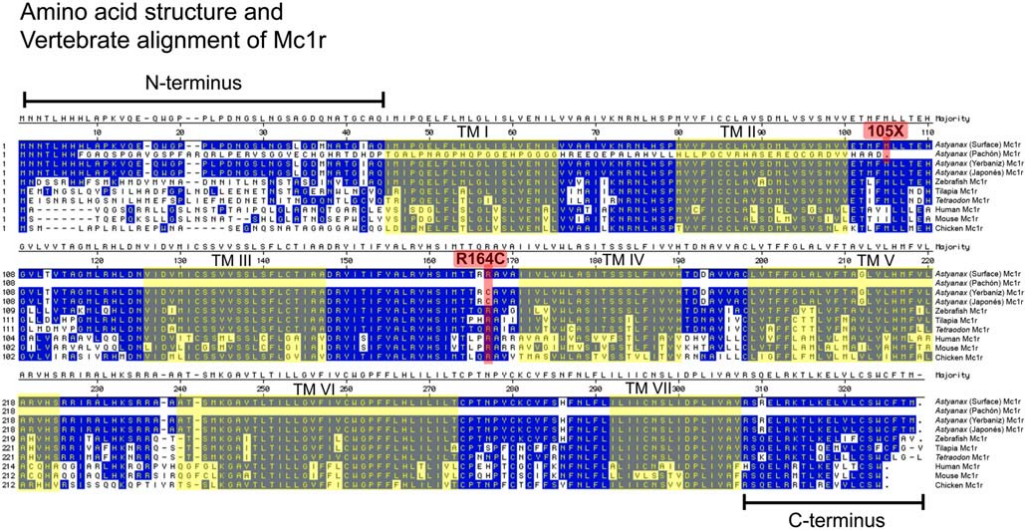
Vertebrate alignment of the amino acid sequence of Mc1r. Mc1r protein sequences of Surface (wild type) and three populations of cave *Astyanax*, three teleost fish species (zebrafish, Tilapia, Tetraodon), and three amniotes (human, mouse, chicken) were aligned using MegAlign 6.1. Conserved protein sequences are depicted in blue, unconserved regions are shown in white. The seven (I–VII) transmembrane domains (TM) are shaded in yellow. The positions of the premature stop codon introduced at position 105 in the Pachón form of Mc1r, and the arginine mutation at position 164 found in individuals derived from the Yerbaniz and Japonés caves are depicted in red. Transmembrane domains are predicted from [66].

To try to identify coding changes that alter Mc1r activity in Pachón cavefish, and hence could be responsible for the brown phenotype, we cloned the entire open reading frame and portions of the 5′ and 3′ UTR from both Surface and Pachón cavefish DNA. Based on predicted protein homology to *Danio rerio*, we identified the predicted start codon, N-terminus, seven transmembrane domains, C-terminus and stop codon in the Surface fish sequence of *Mc1r* (Figure 6). There is no evidence of a non-functional, derived allele in Surface fish when the sequence is compared across multiple teleost and other vertebrate species.

We next compared these sequences to the open reading frame in Pachón cavefish and discovered a 2-base-pair deletion in the extreme 5′ region of the ORF (Figure 3A; positions 23,24). Our sequence analysis predicts this deletion to result in a non-functional transcript as it produces a frame-shift, as well as the introduction of a premature stop codon at nucleotide position 315 (Figure 3A). The resulting protein carries no sequence similarity to the wild type form of the protein, lacking transmembrane domains and C-terminus (Figure 6). Aside from the 2-base-pair deletion, there were no other SNPs or sequence changes evident in the remainder of the ORF sequence of Pachón compared to Surface *Mc1r* sequence. This result, taken together with the genetic mapping data, strongly suggests that the two base pair deletion in the Pachón *Mc1r* allele, could represent the brown mutation.

Complementation tests had indicated that the same gene is responsible for the brown phenotype in several different caves [16]. To determine whether *Mc1r* coding mutations could be identified in other independently derived populations of cavefish, we extended our complete sequence analyses to representative individuals from nine other caves, specifically the Arroyo, Chica, Curva, Japonés, Micos, Molino, Piedras, Tinaja and Yerbaniz populations. Interestingly, the 2-base-pair deletion discovered in members of the Pachón cave was also found, in heterozygous form, in a single member from each of the Yerbaniz and Japonés cave localities (Table 1). Further, with the exception of these two localities, we found no differences in *Mc1r* coding sequence relative to the Surface (wild type) populations. In the Yerbaniz and Japonés caves, however, we discovered the identical point mutation (C490T) causing an arginine to cysteine mutation (R164C) in the second intracellular loop of Mc1r protein (Figures 3B, 6). In addition, we identified a silent mutation (G666A) in Yerbaniz fish that was not present in the Japonés individuals we sampled (Figure 3C). The Yerbaniz and Japonés caves are located within ∼5 km of one another and likely represent the same cave system (perhaps connected through a contiguous underground network). Therefore, functional analyses (see below) were carried out using the R164C mutant allele cloned from a representative member of the Yerbaniz population.

Interestingly, mutations have been described in other species in the Mc1r arginine residue homologous to the one mutated in Yerbaniz and Japonés individuals, including rock pocket mice (*Chaetodipus intermedius*) and humans. In particular, in humans the R160W Mc1r variant (homologous to position 164 in *Astyanax*) is one of two alleles strongly associated with the inheritance of red hair and pale skin [26],[27],[34],[35]. Mutations of this amino acid have been demonstrated to convey diminished receptor function [21], [36]–[38]. Therefore, we reasoned that the identical charge-changing amino acid mutation at the homologous position would be extremely likely to cause diminished function of Mc1r protein in the Yerbaniz and Japonés populations of cavefish, explaining the presence of the brown mutation in these fish.

### Brown Mutants Carrying Two Copies of the Pachón Δ23,24 Allele Demonstrate a Significant Decrease in Melanophore Number and Melanin Content Compared to Wild Type

Prior reports of the brown mutation did not provide detailed descriptions of the depigmentation phenotype exhibited by mutant individuals. The first paper on the subject reported brown mutants “to have melanophores smaller in size and fewer in number” than wild type fish [p. 10 [18]]. Wilkens, 1988 [4] similarly reported a decrease in number and melanin content, depicting scale melanophores derived from Sabinos cave individuals as having much less melanin than surface individuals [p. 305 [4]].

**Figure 7:**
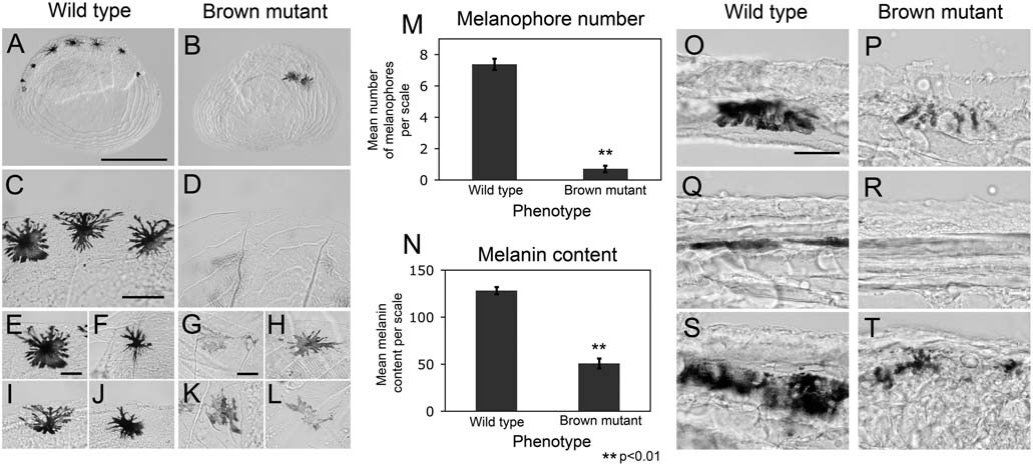
Variation in melanophore number and melanin content in scales derived from Surface (wild type) and brown mutant fish. Individual scales were collected from (A) wild type and (B) brown mutant individuals and assessed for (M) total number of melanophores. (B,D,M) Scales drawn from individuals demonstrating the brown phenotype (carrying two copies of the Pachón Δ23,24 allele) had significantly less numbers of melanophores per scale. (A,C) In wild type scales, melanophores were frequently localized to the periphery of the scale, while (B,D) brown mutant melanophores were rarely at the periphery. Multiple melanophores observed in both (E,F,I,J) wild type and (G,H,K,L) brown mutant scales demonstrated a variety of morphologies, demonstrated by representatives from both phenotypes. (N) The amount of melanin is significantly lower in brown mutant melanophores. Skin tissues derived from the identical region of the dorsal flank demonstrated qualitatively lower amounts of melanin per melanophore in (P,R,T) brown mutants (carrying two copies of the Pachón Δ23,24 allele) compared to (O,Q,S) wild type individuals. Three representative regions were analyzed in each individual. Overall, less melanophores were observed in brown mutant individuals, demonstrated by the absence of pigment in R. Scale bars: A,B = 500 µm; C,D = 100 µm; E–L = 50 µm; O–T = 30 µm.

To have a clearer understanding of the phenotypic consequences of the *Mc1r* mutation we identified, we quantified the number of melanophores present on scales collected from surface and brown mutant individuals (Figure 7A,B), in the region employed in our phenotypic analysis. We found that wild type melanophores derived from surface individuals were more numerous and darker, compared to those of brown mutants (Figure 7A–N). Further, melanophores in wild type scales were most often localized to the distal periphery of the scales (Figure 7C), however were rarely found in the equivalent position in brown mutant scales (Figure 7D). Representative melanophores from wild type and brown mutants were comparable in size, however the amount of melanin present appeared significantly decreased in brown mutant scales (Figure 7E–L). Brown mutant scales demonstrated significantly lower numbers of melanophores compared to wild type (Figure 7M). Further, brown mutants had a significantly lower amount of melanin per melanophore compared to wild type melanophores (Figure 7N).

We also compared melanophores in cryosectioned tissues through the epidermis of the identical region of a representative surface (wild type) and brown mutant individual. These sections were also collected at the same region assayed in our phenotypic analyses (see Methods). We routinely found less (or absent) melanophores and or/melanin in the same regions of brown mutant versus wild type fish. This is likely due to the fact that brown mutants have lower numbers of melanophores than wild type (Figure 7B,M). In equivalent regions where we found melanophores, however, there was far less melanin per cell in brown mutants compared to wild type (Figure 7O–T).

**Figure 8:**
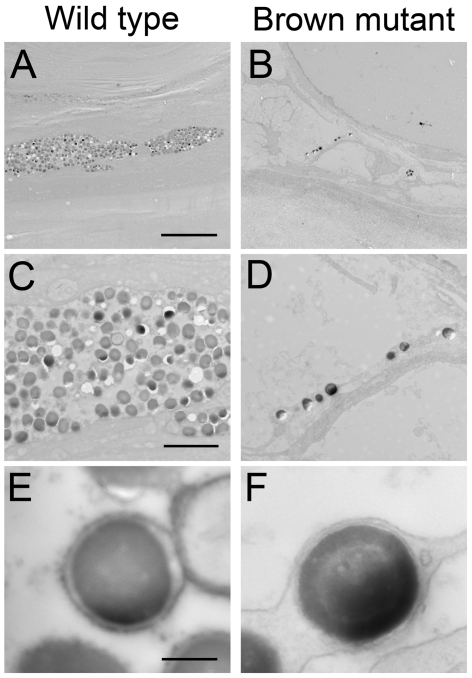
Brown mutant melanophores contain a lower number of melanin granules compared to wild type. Corresponding ultra-thin (95 nm) sections through tail tissue of a representative (A,C,E) Surface and (B,D,F) brown mutant individual (carrying two copies of the Pachón Δ23,24 allele) were processed and imaged using high-resolution electron microscopy. Wild type tissue sections routinely demonstrated densely packed granules of melanin, while sections through brown mutant tissue contained far fewer melanin granules. Overall, melanin granules identified in wild type and brown mutants were the same size and contain comparable amounts of melanin. Scale bars: A,B = 10 µm; C,D = 2.5 µm; E,F = 250 nm.

To determine the basis for decreased melanin in brown mutants, we processed identical regions of tail tissue for electron microscopy. Surface tissue demonstrated rich clusters of granules within melanophores (Figure 8A,C,E), while brown mutant tissue demonstrated infrequent and few clusters of melanin granules (Figure 8B,D,F). Overall, the amount of melanin per granule and the size of melanin granules were comparable between wild type and brown mutant tissues (Figure 8E,F).

### *Mc1r*-Morpholino Knockdown Experimentally Recapitulates the Brown Mutation in Zebrafish

**Figure 9:**
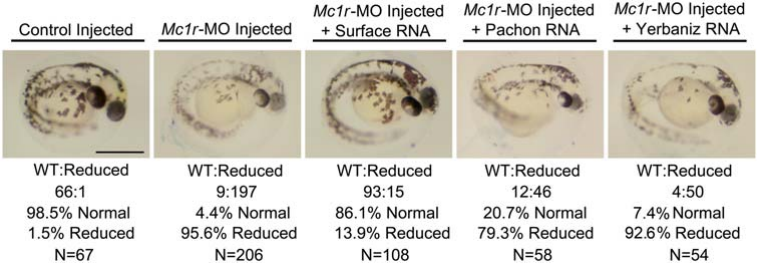
The brown mutation is recapitulated in *Mc1r*-MO knockdown experiments and is rescued by the Surface form of *Mc1r* in the zebrafish. Single-celled zebrafish embryos were injected with 0.2 µM of a MO-oligonucleotide directed against the first 25 base pairs of the *Danio rerio* form of *Mc1r* (see Methods). Compared with embryos receiving a mock injection of 2× 1 nl of Danieaux's solution, embryos receiving MO injections demonstrate significantly reduced pigmentation. This reduced pigmentation phenotype was highly penetrant, occurring in 95.6% of individuals (n = 206) receiving the injection. Rescue experiments were performed in which individuals receiving *Mc1r*-MO injection were co-injected with *in vitro* transcribed RNA from Surface, Pachón, or Yerbaniz constructs. Individuals co-injected with Surface *Mc1r* RNA were rescued from the reduced pigmentation phenotype (86.1%, n = 108). Individuals co-injected with RNA derived from Pachón or Yerbaniz allele failed to rescue the diminished pigmentation phenotype (20.7% in 58 individuals; 7.4% in 54 individuals, respectively). Scale bar: 500 µm.

To demonstrate that decreased functioning of *Mc1r* results in reduced pigmentation, we took advantage of the zebrafish system where techniques of gene knockdown are well established. We first injected zebrafish embryos with a morpholino (MO) targeted to the first 25 base pairs of the *Danio Mc1r* mRNA (Figure 9). Consistent with the well-described role of Mc1r in other vertebrate systems, we found a qualitatively decreased melanin content within melanophores of MO-injected individuals (Figure 9). Further, we observed a corresponding increase in the severity of reduction with increased dosage of MO injection (data not shown).

We also noted a decrease in eye pigmentation compared to same-aged wild type zebrafish recapitulating one of the hallmarks of the brown phenotype (Figure 9). In some cases, we could clearly detect a qualitative decrease in the number of melanophores on the developing yolk sac. However, this trait was highly variable in uninjected controls, suggesting a variation in the number or migration rate of early melanophores on to the developing yolk sac even in control embryos.

Next, we co-injected individuals with *Mc1r* -MO and *in vitro* transcribed RNA derived from the Surface, Pachón or Yerbaniz allele of *Mc1r* (Figure 9). Individuals who were injected with both the MO and Surface RNA had a rescued melanin phenotype in 86.1% of cases (Figure 9). This demonstrates that the Surface form of *Mc1r* is properly translated and expressed in *Danio*, and further, can function normally in place of the endogenous protein. In contrast, individuals who were injected with either the *in vitro* transcribed Pachón or Yerbaniz RNA did not recover normal levels of melanin (compare wild type with 20.7% in Pachón and 7.4% in Yerbaniz, Figure 9). This data provides *in vivo* evidence that the two *Astyanax* cave alleles of *Mc1r*, in contrast to the surface allele, fail to rescue the depigmented phenotype in the zebrafish system.

### The Δ23,24 and C490T Alleles of *Mc1r* Are Restricted to the Pachón and Yerbaniz/Japonés Caves

To estimate the frequency of the variant *Mc1r* alleles identified in this study, we sampled and genotyped a total of 140 individuals from 12 different cave localities, and a total of 231 individuals from 13 different surface localities (Table 1, Figure 1). Every individual collected from the Pachón cave population were homozygous for the Δ23,24 allele. The majority of individuals sampled from the Yerbaniz (n = 9) and Japonés (n = 8) cave populations were homozygous for the C490T allele. One individual sampled from each cave, however, were heterozygous for the C490T allele and the Δ23,24 allele. No individual from any of the surface populations carried the Δ23,24 or C490T allele.

Our sampling does indicate that both variant alleles of *Mc1r* are extremely rare outside of the Pachón, Yerbaniz and Japonés caves and are not polymorphisms in the current river population. However, an important caveat is that the frequency of alleles currently found in the surface fish population may not reflect the allelic frequencies from the time when the surface fish colonized the caves. Indeed, it is extremely likely that at least the Δ23,24 allele was present in the ancestral surface population and did not arise as a *de novo* mutation in the caves, since the same two-base pair mutation was identified in the Yerbaniz and Japonés caves. The Pachón and linked Yerbaniz/Japonés caves, which are approximately 35 km apart, do not appear to be connected geologically and moreover molecular analyses indicate that different microsatellite alleles were fixed in the two caves at all loci examined. If, indeed, the Δ23,24 allele was maintained in the two caves independently and assuming that this allele was relatively rare in the ancestral population as it is in the current surface population, it would at least raise the possibility that the brown mutation was positively selected in the two caves, even though the adaptive significance of the loss of *Mc1r* is less than obvious.

## Discussion

### Identification and Characterization of *Mc1r* Mutations in a Wild Population of Teleost Fish

Numerous studies have characterized *Mc1r* in an extensive variety of model systems [29], [39]–[41] and domesticated species [42]–[45]. More recently, the role of *Mc1r* variants in the adaptive coloration in several natural populations has been demonstrated [24], [46]–[50]. In human beings, the role of various *Mc1r* variants has similarly been previously explored, particularly those showing strong associations to skin cancer development [51]–[53]. In this study, we identify two coding alterations to the gene *Mc1r* that presumably destroy normal functioning of the receptor protein. These are the first examples of coding mutations in *Mc1r* reported in a wild species of fish. Furthermore, this study implicates *Mc1r* in the novel role of depigmentation in wild animals populating a cavernous environment.

The 2-bp deletion discovered in Pachón cavefish is predicted to cause a frame-shift leading to the introduction of a premature stop codon at position 315. This allele is likely to be amorphic, and the truncated protein encoded by this locus to be non-functional. The mutation discovered in populations of Yerbaniz and Japonés individuals, C490T, causes a cysteine substitution at position R164, which is homologous to the R160W mutant in human individuals (Figure 6). This point mutation, in two copies, has been reported to cause loss of Mc1r receptor function in humans [26],[54]. In addition, the *Mc1r* null phenotype in humans is red hair and pale skin [55], a phenotype similar to individuals harboring the R160W mutation.

Thus, we believe the allele identified in members of the Yerbaniz and Japonés caves is also amorphic. Consistent with this interpretation, the brown phenotype is similar in the Pachón and Yerbaniz caves [16]. Morpholino knockdown studies in zebrafish further imply these to be non-functional alleles since neither Pachón nor Yerbaniz forms of *Mc1r* are capable of rescuing diminished pigmentation in MO-injected individuals (Figure 9). The phenotype we observe in these studies does produce a phenotype very similar to what one would predict from loss-of-function alleles of *Mc1r* in teleost fish. This claim of complete abrogation of Mc1r function remains speculative until a time when these receptors can be tested *in vivo* (e.g., gene deletion studies) for the possible presence of residual function.

The decrease in eye pigmentation, previously described as part of the brown phenotype and recapitulated in our zebrafish MO injections differs, however from what is seen in mammals, where altered Mc1r functioning does not appear to affect eye color [26]. Further, *Astyanax* carrying the brown mutation and *Mc1r* zebrafish morphants demonstrate a decrease in the total pigmentation of melanophores as well as pigment distribution within cells (Figure 9). This phenotype differs from the established role for α-MSH (and the inferred role for Mc1r) in mediating the distribution of melanin within melanocytes [reviewed in [21]]. These results suggest that there are differences in the function of the Mc1r receptor between higher and lower vertebrates.

### Regressive Evolution of Pigmentation

Regressive changes are defined as those traits that decrease or degenerate within a lineage over the course of evolution [56],[57]. Specifically, the loss of pigmentation in *Astyanax* cave morphs is frequently cited as an example of regressive evolution [5],[16],[58],[59]. For the brown trait to be considered regressive, it must be expressed in the wild (i.e., not be a molecular alteration that only manifests itself as a visible phenotype under the artificial conditions of crosses set up in the laboratory). In this study, albino individuals drawn from the wild were used in all crossing experiments. Therefore, in our pedigrees, albinism is epistatic to the brown mutation. However, it is clear from the literature that, in at least some caves, the brown phenotype is a true regressive trait.

Sadoglu and McKee, 1969 [18] performed a series of intercrosses of wild-caught Pachón cave individuals. A pedigree of 340 offspring derived exclusively from wild-caught Pachón×Pachón crosses included 233 (68.5%) individuals with the brown phenotype, 107 (31.5%) albino individuals and no individuals with black (wild-type) coloration [p. 11 [18]]. While this may be a limited sample size and hence the absolute frequencies may be open to question, this result clearly demonstrates that not all of the fish in the Pachón cave were at that time albino. Moreover, additional expeditions during the 1960s confirmed that albinism was not present (i.e., not fixed) in all individuals derived from the Pachón cave [13],[60]. Even if the brown mutation was not completely fixed in the population, as in the data set reported by Sadoglu and McKee, 1969 [18], at some frequency the brown mutation would be found in non-albino fish and hence the regressive trait would be expressed.

In contrast, it is more difficult to determine whether the C490T mutation present in Yerbaniz and Japonés individuals arose as a regressive trait. Fish in the Yerbaniz [4],[16],[19] and Japonés [1] caves are reported as albino. Since it is impossible to determine whether the mutations in *Oca2* or *Mc1r* arose first and/or were fixed first, the brown mutation in the Yerbaniz and Japonés caves may have arisen amidst the background of epistatic mutations causing albinism. In this scenario, the brown phenotype would not have been expressed and hence the trait would not be properly termed as regressive. Most likely, in this case, the brown coding alteration would have arisen by neutral mutation and drift.

There are other caves, however, where the brown phenotype is clearly regressive. There are only three independent cave populations in this system that are reported to be albino (Molino, Pachón and Yerbaniz/Japonés). Therefore, the brown phenotype would not be masked in caves that failed to complement the brown mutation such as in fish from the Piedras or Chica caves, where albinism has not been reported (Figure 1).

### The *In Vivo* Role of the Mc1r Receptor in Teleost Fish

There has, thus far, been no description of a loss-of-function mutation in a teleost fish. The phenotype we describe following morpholino knockdown of *Mc1r* closely resembles that seen in Pachón, Yerbaniz and Japonés individuals carrying the coding mutations described here. Moreover, co-injection of the Δ23,24 or C490T alleles did not rescue the morphant phenotype. We therefore strongly suspect this is the null phenotype in teleosts. However, since the knockdown could be incomplete, in principle, it would be worth considering what might be learned about the role of the *Mc1r* gene from comparison to other species.

One might expect some level of functional conservation between higher and lower vertebrates. For example, just as in amniotes, Mc1r is expressed in the skin and its associated structures (i.e., scales) of fish [61],[62]. Additionally, numerous analyses have identified a single orthologous copy of the gene *Mc1r* in birds, mammals and fish species [63]–[66]. Further, the high degree of amino acid conservation of the Mc1r receptor across vertebrates (∼400 million years of evolution) suggests an essential role for Mc1r in vertebrate physiological function [63],[66].

Currently, data on the null phenotype for *Mc1r* is only available for two species, mice and humans. The classical mouse mutant, extension, carries two non-functional copies of *Mc1r* [23]. The phenotype of this mouse is a yellow coat, however, some residual eumelanin synthesis in the fur of this mouse has been reported [67]. In contrast, the phenotype of a homozygous *Mc1r* null human individual is red hair and fair skin [55]. Therefore, the expression of *Mc1r* loss-of-function alleles in different animals results in slightly different phenotypes. Moreover, greater caution must be maintained in extrapolating from the mammalian mutants to teleost fish since there are important differences in the role of the Mc1r pathway in these taxa.

In mammals, the Mc1r ligand, α-MSH, stimulates *de novo* melanin synthesis in melanocytes [40],[68]. The function of α-MSH in fish has most frequently been attributed to inducing rapid dispersal of melanin granules from clustering around the nucleus to dispersal throughout the cell body and dendritic extensions of the melanophores [61],[63],[64],[69]. This role correlates extremely well with the phenotype we observe in the brown individuals at the ultrastructural level. In the wild, this rapid redistribution of melanin grants fish the ability to adapt their color to the surrounding substrate [63].

There are also significant differences in the physiology of pigmentation between amniotes and fish. For example, mammals have a single pigment cell type, the melanocyte, capable of producing black, brown, red or yellow pigment [70]. Normal signaling of the hormone, α-MSH, acting through Mc1r on melanocytes induces cells to produce black or brown eumelanin; while attenuated signaling results in production of red or yellow phaeomelanin [reviewed in [71]]. In contrast, teleost fish have three types of pigment-producing cells (chromatophores): melanophores, xanthophores and iridophores [70]. Further, fish melanophores contain only eumelanin and do not produce phaeomelanin, the primary pigment produced in mouse and human *Mc1r*-null individuals [69].

Thus, a cross-species comparison likely sheds more light on the range of effects Mc1r can have on pigmentation than on the probable null phenotype in teleosts. Although the mutants we observe in the Pachón and Yerbaniz/Japonés caves are likely to be null, this remains to be confirmed.

### Drift or Selection of the Brown Phenotype?

The question of how regressive phenotypes arise in nature has long interested biologists. The fascinating examples of parallelism provided by cave animals have stimulated a rich history of hypotheses on the evolution of cave phenotypes stretching back to Charles Darwin, who observed: “it is difficult to imagine that eyes, although useless, could be in any way injurious to animals living in darkness”, and therefore attributed loss of cave form phenotypes to “disuse” [72]. Two predominant theories seek to explain the regression of characters in cave animals: neutral mutation and selection [56],[73],[74]. According to the neutral mutation hypothesis, a particular trait that is no longer under selection is free to drift and accumulate mutations [4]. This hypothesis is tied closely to whether the trait plays an important ecological role. In fish, the primary function of Mc1r is to mediate distribution of melanin granules in fish melanophores, allowing the organism to adapt to its background substrate [63]. The cave environment includes the complete absence of light, and therefore this trait is rendered useless. Therefore, in the absence of phenotypic consequences, the function of Mc1r is likely no longer under selection. Thus, the most probable scenario in this case may be that, following colonization into the cave, the pigmentation function of Mc1r became free to accumulate mutations via drift.

Nonetheless, as noted above, the presence of Δ23,24 allele in both cave populations could indicate that a preexisting mutation was independently selected in the two caves. In this case Mc1r may have a currently unappreciated pleiotropic effect that, although perhaps relatively small, is sufficient to provide a selection in the cave environment. If so, the C490T mutation is likely to confer the same phenotypic effect, as all fish in the Yerbaniz/Japonés cave we sampled carry two mutant Mc1r alleles, but neither of the two mutant alleles has become fixed under selection.

In amniotes living above ground, the selection on the *Mc1r* locus is clearer. In many cases, alterations of Mc1r functioning lead to phenotypic consequences for coloration (i.e., coat color in mammals, plumage color in birds). These phenotypic differences are presumably selected for their adaptive functions for crypsis [46] or sexual ornamentation [75]. In humans, *Mc1r* variation presents a confusing picture. The phenotypic effects caused by variation at the *Mc1r* locus in non-African populations have been explained as a consequence of sexual selection [76], as well as drift [77], [reviewed in [78]]. Genome-wide analyses, however, failed to find strong evidence for selection at *Mc1r* locus in humans [79].

### Evolution of Pigmentation Alterations in Teleost Fish

Regressive phenotypes have long been of interest to evolutionary biologists, given the many examples of regressive (degenerate) phenotypic variation when comparing humans to their primitive ape-like ancestors, e.g., hair loss [80]. While many theories seek to explain the process of phenotypic degeneration, genetic insights into the underlying bases of these traits has remained elusive. Here, we describe the genetic basis of one of the earliest described genetic traits in the blind Mexican cave tetra, the brown mutation. We have found coding sequence modifications in two independent cave forms, the Pachón and Yerbaniz/Japonés caves, that explain the reduced function of the 7-transmembrane domain receptor protein, Mc1r.

Complementation tests indicate that other cave populations of *Astyanax*, including Piedras and Curva, have brown phenotypes caused by mutations at the same locus as in Pachón and Yerbaniz. We failed to find any coding alterations in *Mc1r* sequence from fish collected from these other caves, suggesting that these populations likely carry regulatory mutations leading to a decrease or loss of Mc1r activity (Figure 1).

The genetics of pigmentation have been explored in several model teleost fish including zebrafish [81],[82], medaka [83] and fugu [84]. More recently, the evolution of pigment variation in natural populations of sticklebacks has been explored using linkage analysis [85]. These authors discovered the relevant locus controlling lighter pigmentation to be due to cis-regulatory changes affecting expression of the gene, *kit ligand* (*kitl*). Further, it was discovered that evolution in the cis-regulatory regions of the *kitl* locus similarly appears to control the evolution of lighter skin in recent humans [85],[86]. Interestingly, another genetic locus examined in the zebrafish *golden* mutant (*SLC24A5*) appears to also play a role in pigmentation evolution in recent humans [86],[87]. Mutations in *Mc1r* likewise are found in humans in addition to *Astyanax* and other species. Thus, there seems to be a common tool-kit of pigmentation genes used to modify coloration during evolution of widely divergent taxa.

The participation of *Mc1r* in the evolution of *Astyanax* cavefish depigmentation clarifies the essential role of this gene in vertebrates. Its role in the regressive evolution of pigmentation in independently derived cavefish indicates that certain genes may be important loci for convergent evolution of specialized traits of cave-adapted animals.
